# An exploratory study of associations between the ICD-11 personality disorder model and eating pathology

**DOI:** 10.1186/s40337-022-00658-y

**Published:** 2022-08-31

**Authors:** Johannes Stricker, Friederike Barthels, Romina Müller, Reinhard Pietrowsky

**Affiliations:** Psychology Department, Heinrich Heine University Düsseldorf, Universitätsstraße 1, 40225 Düsseldorf, Germany

**Keywords:** Personality dysfunction, Personality traits, Eating pathology, International Classification of Diseases (ICD-11), Dimensional assessment

## Abstract

**Background:**

Recently, the International Classification of Diseases 11th Revision (ICD-11) has introduced a paradigm shift in personality disorder conceptualization. The novel ICD-11 personality disorder model comprises a dimensional assessment of personality dysfunction and five maladaptive personality trait domains. Maladaptive personality plays a central role in eating pathology. Yet, relations between the ICD-11 personality disorder model and eating pathology are, to date, unclear. Thus, this study aimed to explore the bivariate, incremental, and interactive associations of the ICD-11 personality disorder model components with eating pathology domains.

**Methods:**

A predominantly female (85%) sample of 888 German-speaking community adults completed validated self-report measures of personality dysfunction, the ICD-11 personality trait domains, and five eating pathology domains (drive for thinness, bulimia, body dissatisfaction, orthorexia, binge eating). Bivariate and hierarchical regressions models were used to investigate bivariate, incremental, and interactive relations between the ICD-11 personality disorder model components and eating pathology.

**Results:**

Personality dysfunction and the ICD-11 personality trait domains showed statistically significant bivariate relations with eating pathology. Additionally, personality dysfunction and most ICD-11 personality trait domains displayed incremental links with eating pathology. Finally, the relations of the ICD-11 personality trait domains with eating pathology were largely independent of the severity of personality dysfunction.

**Conclusions:**

This study indicated that all ICD-11 personality disorder model components are uniquely linked to eating pathology. Beyond maladaptive personality trait domains, the strong and incremental relations of personality dysfunction with eating pathology have potential implications for theory building. Further research using longitudinal designs is needed to evaluate causal links between the ICD-11 personality disorder model components and eating pathology.

**Supplementary Information:**

The online version contains supplementary material available at 10.1186/s40337-022-00658-y.

## Plain English Summary

The International Classification of Diseases 11th Revision (ICD-11) contains a novel personality disorder model. We explored how this model’s components (i.e., personality dysfunction, five maladaptive personality traits) relate to eating pathology. The model components were significantly and complementarily related to a drive for thinness, bulimia, body dissatisfaction, orthorexia, and binge eating. Hence, the ICD-11 personality disorder model may potentially prove helpful in better understanding individual differences in eating pathology.

**Supplementary Information:**

The online version contains supplementary material available at 10.1186/s40337-022-00658-y.

## Background

Personality is crucial for the onset, course, and treatment of eating pathology (for reviews, see [1–4]. Much of the previous research into the personality-eating pathology association has used categorical conceptualizations (i.e., presence vs. absence of an eating or personality disorder; e.g., [5]). Yet, recently, dimensional models of personality disorder and eating pathology have been increasingly advocated [6, 7].

Perhaps most prominently, the novel ICD-11 personality disorder model takes a dimensional and trait-based approach, comprising general personality dysfunction and five more specific personality trait domains [8, 9]. Although the ICD-11 will be used in clinical practice worldwide and despite the importance of personality for case formulations and personalized treatment of eating disorders [4, 10], the relation of the ICD-11 personality disorder with eating pathology is, to date, unclear.

This study investigated the associations of the ICD-11 personality disorder model components with a broad spectrum of eating pathology. More precisely, in a general population sample, we assessed (i) how the ICD-11 personality disorder components are bivariately related to eating pathology, (ii) whether both ICD-11 personality disorder model components (personality dysfunction, trait domains) are incrementally related to eating pathology, and (iii) whether personality dysfunction moderates the relation of the ICD-11 personality trait domains with eating pathology.

### The ICD-11 personality disorder model

The ICD-11, an authoritative mental disorder classification system, distinguishes two components of personality disorder: personality dysfunction and maladaptive personality trait domains [8, 9, 11]. In the first step, clinicians are instructed to indicate the general severity of persistent intra- and interpersonal problems (personality dysfunction; for recent reviews, see [12–14]). In the second step, any number of five personality trait domains (negative affectivity, detachment, dissociality, disinhibition, anankastia) can be used to more precisely describe the respective personality pathology [9]. Negative affectivity describes the tendency to be prone to experiencing negative emotions. Detachment describes the tendency to remain interpersonally and emotionally distant. Dissociality describes the tendency to disregard the feelings and rights of others. Disinhibition describes the tendency to act impulsively without considering potential negative consequences. Finally, anankastia describes the tendency to focus narrowly on one’s rigid standards [9].

Conceptually, the ICD-11 assumes that personality dysfunction and maladaptive traits are distinguishable. Whereas personality dysfunction captures broad inter-and intrapersonal deficits (indicating the *severity* of personality problems), maladaptive traits capture more specific cognitive, behavioral, and affective patterns (indicating the *style* of personality problems) [9, 15, 16]. More pronounced maladaptive personality traits may contribute to personality dysfunction, but according to the ICD-11, trait domains and personality dysfunction are not identical [9]. For example, a person with heightened anankastic behaviors may either cope relatively well (high anankastia, low personality dysfunction) or experience constant self-criticism and interpersonal conflict (high anankastia, high personality dysfunction).

An emerging body of research has investigated the distinguishability of personality dysfunction and personality traits empirically. In previous studies, the ICD-11 personality trait domains were mostly positively associated with personality dysfunction, with correlations ranging, e.g., from *r* = −0.09 for anankastia to *r* = 0.63 for negative affectivity [17]. The magnitude of these bivariate associations does not indicate redundancy between the ICD-11 personality trait domains and personality dysfunction. Recent work has also investigated the incremental (unique) utility of both ICD-11 personality disorder model components for statistically explaining relevant outcomes. In one study, personality dysfunction and the ICD-11 personality trait domains were incrementally related to stress, anxiety, and depression beyond the respectively other ICD-11 personality disorder model component [18]. However, other studies found mixed results regarding the incremental utility of traits and personality dysfunction (e.g., [19]). As the ICD-11 personality disorder model is currently entering global clinical practice [20–22], its links with relevant psychopathology domains are gaining increasing attention (e.g., [18, 23, 24]). Yet, to date, no study has investigated associations of the ICD-11 personality disorder model with eating pathology.

### The dimensional assessment of eating pathology

Eating pathology encompasses different dysfunctional eating and weight-related attitudes and behaviors. Traditionally, eating pathology has been assessed categorically, which is often a prerequisite in clinical practice. However, dysfunctional eating-related behaviors, cognitions, and emotions also contribute to distress in persons not meeting the criteria for a mental disorder diagnosis (e.g., [25, 26]). Additionally, persons with eating disorders frequently display other comorbid mental disorders [27], complicating the identification of correlates specific to eating pathology. Consequently, dimensional approaches that may capture sub-threshold to severe symptomatology and decompose eating pathology into more specific symptom domains have been increasingly supported in recent years [7]. Using dimensional assessments and differentiating eating pathology domains has been specifically recommended for investigating eating pathology-personality associations [28].

### Linking the ICD-11 personality disorder model and eating pathology

Various theoretical models suggest strong ties between personality and eating pathology (as risk factors, predisposing factors, correlates, consequences, or complications of each other) (e.g., [29, 30]). Based on these solid conceptual links, a large number of empirical studies have explored relations between personality and eating disorders [1, 2]. Many of these previous studies have investigated comorbidities of disturbed eating and personality disorders (for a meta-analysis, see [5]). Other studies have compared mean levels of personality traits between persons with and without eating disorders. For example, these studies often found increased neuroticism (conceptually related to ICD-11 negative affectivity) and, to a lesser extent, decreased extraversion (conceptually related to ICD-11 detachment) in samples with different eating disorders (see [2]). Findings on agreeableness (conceptually related to lower ICD-11 dissociality) have been mixed (e.g., [31]). Impulsivity (conceptually related to ICD-11 disinhibition) has been sometimes linked to bulimia (e.g., [32]) and frequently linked to binge eating (e.g., [33]). Obsessive–compulsive traits (conceptually related to ICD-11 anankastia), such as perfectionism, are thought to play a pivotal role in the transdiagnostic drive for thinness and body dissatisfaction (e.g., [34]) and in orthorexic behaviors (e.g., [35]).

One important limitation of most previous work linking maladaptive personality to eating pathology is that previous categorical personality disorder models were unable to differentiate the effects of general personality dysfunction, specific maladaptive traits, and acute distress associated with the presence of personality pathology. In contrast, using the novel ICD-11 personality disorder model and dimensional measures allows more differentiated and fine-grained insights into links of problematic personality with eating pathology domains.

Thus far, studies on links between current dimensional models of personality disorder with eating pathology have been limited to the DSM-5 Alternative Model of Personality Disorder (AMPD) [36]. In one study, orthorexic eating behavior was positively correlated with DSM-5 detachment, antagonism, and psychoticism in a general population sample [37]. In a different study, using theoretically informed models, some facets conceptualized below the DSM-5 AMPD trait domain-level correlated with different eating pathology domains in community adults: the rigid perfectionism facet with restricted eating and the impulsivity and anxiousness facets with binge eating [28]. To date, no study has investigated links between the ICD-11 personality trait domains and personality dysfunction with eating pathology.

Personality dysfunction accounts for a large proportion of the overlap between maladaptive personality traits and their links with other psychopathology [19]. Consequently, it has been debated whether personality dysfunction and more specific maladaptive personality trait domains may be redundant in current personality disorder models (see, e.g., [16, 38, 39]). To date, it is unclear whether associations between maladaptive personality traits and eating pathology are due to *general* personality dysfunction (see, e.g., [14, 15]) or *trait-specific* characteristics. Thus, clarification of the *incremental* associations of maladaptive traits and personality dysfunction with eating pathology is required.

Another question regarding the relation of the ICD-11 personality disorder model with eating pathology concerns potential interactive effects. Recent research suggests that personality dysfunction moderates associations of maladaptive traits with relevant behaviors and outcomes [40, 41]. For example, trait-specific behaviors and thought patterns (e.g., rigid standards) may be indicative of more pronounced eating pathology in persons with high personality dysfunction (e.g., be problematic in the presence of identity and self-worth problems) but unproblematic in persons with low personality dysfunction (e.g., persons with stable self-worth). The ICD-11 suggests to first assess personality dysfunction and then maladaptive personality trait domains [9]. If personality trait domain-eating pathology associations hinged on the presence of personality dysfunction, trait domain-level assessments might only be sensible following a preceding personality dysfunction screening. Hence, it is important to clarify whether personality dysfunction moderates personality trait domain-eating pathology links.

### The current study

The ICD-11 introduces a paradigm shift in personality disorder conceptualization, affecting future clinical practice and research. To date, the associations between the ICD-11 personality disorder model and eating pathology are unclear. Thus, this study investigated the bivariate, incremental, and interactive relations of the ICD-11 personality disorder model components with a broad spectrum of eating pathology in a large general population sample. As this is the first study investigating links between the ICD-11 personality disorder model and eating pathology, we did not preregister specific hypotheses.

## Methods

### Sample and procedure

We recruited German-speaking community adults via different social media channels (e.g., various Facebook groups), websites (e.g., of a German popular science magazine), and flyers (e.g., on-campus and in supermarkets). The study was advertised as a survey on eating behaviors and personality. All adults (i.e., ≥ 18 years of age) with sufficient proficiency in German were eligible to participate. There were no other inclusion or exclusion criteria. The participants completed all questionnaires online in *Qualtrics*, a web-based survey software. To screen for careless responders, we included two instructed response items (e.g., “To ensure the data quality, please select the leftmost response option for this statement (‘strongly disagree’).”). We excluded 29 participants for failing to solve these instructed response items correctly. The final sample comprised 888 German-speaking community adults. Table 1 displays the detailed sample characteristics. All participants provided their informed consent. The study was performed following the ethical guidelines of the German Society for Psychology. For low-risk studies (questionnaire studies in general population adults), no additional approval from an institutional review board is required in Germany. Due to the explorative nature of this study, we did not determine the sample size a priori. Instead, we terminated data collection after a predefined period of one month. Our code and data are available via the Open Science Framework: https://osf.io/ybwu9/?view_only=81f7f8794bbf438790c5e58ab829794b.

**Table 1:** Sample characteristics

Age (years), *M*	35.26 (*SD* = 12.83, range = 18 to 75)
Gender, *n* (%)	
Female	755 (85%)
Male	124 (14%)
Other	8 (1%)
Not disclosed	1 (< 1%)
Highest educational qualification	
High school degree	569 (64%)
University or college degree	318 (36%)
Not disclosed	1 (< 1%)
Occupational status	
Employed or self-employed	476 (54%)
Students or trainees	287 (32%)
Currently not working (e.g., unemployment, retirement)	120 (14%)
Not disclosed	5 (< 1%)
Personality dysfunction, *M* (*SD*)	24.24 (6.31)
Negative affectivity, *M* (*SD*)	34.44 (8.17)
Detachment, *M* (*SD*)	26.56 (8.00)
Dissociality, *M* (*SD*)	24.21 (6.35)
Disinhibition, *M* (*SD*)	25.76 (6.58)
Anankastia, *M* (*SD*)	39.11 (6.66)
Drive for thinness, *M* (*SD*)	21.19 (8.92)
Bulimia, *M* (*SD*)	13.83 (6.19)
Body dissatisfaction, *M* (*SD*)	32.51 (12.04)
Orthorexia, *M* (*SD*)	18.13 (5.50)
Binge eating, *M* (*SD*)	27.85 (8.93)

*M* = mean, *SD* = standard deviation

### Measures

#### Personality dysfunction

We used the LPFS-BF 2.0 (German version) [42, 43] to assess personality dysfunction. This self-report instrument comprises 12 items (e.g., “I often do not understand my own thoughts and feelings”) capturing basic impairments underlying personality dysfunction on a 4-point Likert scale ranging from 1 “does not apply at all” to 4 “fully applies”. Higher LPFS-BF 2.0 scores indicate higher personality dysfunction. The LPFS-BF 2.0 has initially been developed for assessing the level of personality dysfunction as described in the DSM-5 AMPD. To date, the LPFS-BF 2.0 is widely accepted as a measure of ICD-11 personality dysfunction (e.g., [23]), as it empirically and conceptually converges with other measures of ICD-11 personality dysfunction (e.g., [24]). Various studies have demonstrated the reliability (e.g., [44]) and validity (e.g., [45]) of the LPFS-BF 2.0.

#### ICD-11 personality trait domains

We used the PiCD (German version) [46, 47] for assessing the ICD-11 personality trait domains. This self-report instrument assesses the five ICD-11 personality trait domains (negative affectivity, detachment, dissociality, disinhibition, anankastia) with 12 items each (e.g., “I am usually an anxious person” for negative affectivity, “I prefer to stay away from other people” for detachment, “I am always ready for conflict” for dissociality, “I tend to act impulsively” for disinhibition, and “I strive for perfection” for anankastia). The PiCD uses a 5-point Likert scale ranging from 1 “strongly disagree” to 5 “strongly agree”. Higher scores in the PiCD trait domain scales indicate more pronounced characteristics of the respective ICD-11 personality trait domain. Various studies have demonstrated the reliability (e.g., [48]) and validity (e.g., [49]) of the PiCD.

#### Eating pathology

We used three self-report measures to assess a broad spectrum of eating pathology. First, we administered three subscales of the Eating Disorder Inventory-2 (EDI-2; German version) [50, 51] that assess problematic eating-related behaviors, cognitions, and emotions: The drive for thinness subscale (7 items), the bulimia subscale (7 items), and the body dissatisfaction subscale (9 items). The drive for thinness scale assesses overconcern with dieting or weight and fear of weight gain. The bulimia subscale assesses binge eating with compensatory behaviors. Finally, the body dissatisfaction subscale assesses dissatisfaction with one’s physical appearance. The EDI-2 subscales use a 6-point Likert format ranging from 1 “never” to 6 “always”. Higher scores in the EDI-2 subscales indicate more pronounced symptoms in the respective eating pathology domain. Various studies support the reliability and validity of the German EDI-2 (e.g., [52]).

Second, we used the Düsseldorf Orthorexia Scale (DOS) [53, 54] to assess excessive fixation on health-conscious eating behavior (orthorexia) with 10 Likert items (e.g., “I can only enjoy eating foods considered healthy”) ranging from 1 “does not apply to me” to 4 “applies to me”. Higher DOS scores indicate more pronounced orthorexia symptoms. The DOS has displayed satisfactory reliability and validity in various studies (e.g., [55]).

Third, we used the Binge Eating Scale (BES) [56] to assess behavioral, cognitive, and emotional features of binge eating (i.e., recurrent episodes of uncontrolled overeating). The BES comprises 16 items with three to four response options, coded as “0” to “2″ or “3″, respectively (e.g., ranging from” I don’t feel any guilt or self-hate after I overeat” to “Almost all the time I experience strong guilt or self-hate after I overeat”). Higher BES scores indicate more pronounced binge eating symptoms. For use in our study, two psychological researchers with high proficiency in English and German independently translated the BES items to German and resolved any discrepancies by discussion. Next, a third psychological researcher back-translated all items. Again, any discrepancies were discussed, and the German scale was refined until a joint agreement was reached. Different studies demonstrate the reliability and convergent validity of the English version of the BES (e.g., [57–60]).

### Statistical analyses

As preliminary analyses, we evaluated the internal consistency (α) and the mean levels of all variables. Additionally, we computed the proportion of variance in eating pathology domains statistically explained by all ICD-11 personality disorder model components jointly. To assess bivariate relations between the ICD-11 personality disorder model components and eating pathology, we conducted a series of bivariate regressions. In each of these bivariate regressions, one component of the ICD-11 personality disorder model predicted one of the eating pathology domains. To assess the incremental relations of each ICD-11 personality trait domain with eating pathology beyond personality dysfunction, we conducted a series of hierarchical regressions. In these analyses, each eating pathology domain was predicted by personality dysfunction in Step 1 and personality dysfunction and one of the ICD-11 personality trait domains in Step 2. Additionally, we conducted hierarchical regressions testing whether personality dysfunction incrementally explains eating pathology beyond the five ICD-11 personality trait domains.

To assess interactive effects of personality dysfunction and the ICD-11 personality trait domains, we entered the personality dysfunction*trait interaction in a third step beyond the main effects of one of the ICD-11 personality trait domains and personality dysfunction in the aforementioned series of hierarchical regressions. Due to the many conducted statistical tests and in line with current recommendations [61], we used *p* < 0.005 as a conservative cut-off criterion for statistical significance in all analyses.

## Results

### Preliminary analyses

Additional file 1: Table S1 displays the bivariate correlations, means, standard deviations, and internal consistencies for all variables. The EDI-2 subscale mean scores obtained in this study were elevated compared to the norm scores from a sample of healthy women [51] (approx. corresponding to the 70th percentile of the norm scores for drive for thinness, approx. 85th percentile for bulimia, and approx. 60th percentile for body dissatisfaction). The DOS mean score (18.13) was similar to the mean score observed in a general population sample in the DOS validation study (17.75) [53]. The PiCD trait domain mean scores were largely comparable to the scores obtained from a general population sample in the validation study of the German PiCD [47]. The LPF-BF 2.0 mean score (24.24) was slightly elevated compared to the mean score obtained from an online community sample in the validation study of the German LPFS-BF 2.0 (22.72) [42]. The internal consistency (α) was satisfactory for all scales (range = 0.78 to 0.93, *Mdn* = 0.85). Jointly, the ICD-11 personality model components statistically explained 18% of the variance in drive for thinness, 22% of bulimia, 12% of body dissatisfaction, 10% of orthorexia, and 25% of binge eating (see Additional file 1: Tables S2 to S7).

### Bivariate relations

Table 2 displays the bivariate relations of the ICD-11 personality disorder model components with eating pathology. Personality dysfunction was related to all eating pathology domains, with regression coefficients ranging from β = 0.19 for orthorexia to β = 0.46 for binge eating (all *p*s < 0.001). Also negative affectivity was related to all eating pathology domains (ranging from β = 0.24 for orthorexia to β = 0.41 for binge eating; all *p*s < 0.001). Detachment was associated with more pronounced body dissatisfaction (β = 0.13, *p* < 0.001) and bulimia (β = 0.10, *p* = 0.004). Dissociality displayed small to medium bivariate relations with all eating pathology domains (ranging from β = 0.09 for body dissatisfaction to β = 0.19 for binge eating, all *p*s ≤ 0.0046). Disinhibition was related to all eating pathology domains (ranging from β = 0.10 for drive for thinness to β = 0.25 for binge eating, all *p*s ≤ 0.003), except orthorexia (β = −0.07, *p* = 0.038). Finally, anankastia was bivariately related to orthorexia (β = 0.21, *p* < 0.001) and drive for thinness (β = 0.13, *p* < 0.001).

**Table 2:** Bivariate associations of the ICD-11 personality disorder model components and eating pathology

ICD-11 personality model disorder component	Eating pathology				
	Drive for thinness	Bulimia	Body dissatisfaction	Orthorexia	Binge eating
Personality dysfunction	0.37*	0.42*	0.33*	0.19*	0.46*
Negative affectivity	0.38*	0.39*	0.27*	0.24*	0.41*
Detachment	0.07	0.10*	0.13*	0.08	0.09
Dissociality	0.12*	0.18*	0.09*	0.13*	0.19*
Disinhibition	0.10*	0.24*	0.13*	−0.07	0.25*
Anankastia	0.13*	0.05	0.07	0.21*	0.04

All values represent standardized regression coefficients. *p < 0.005

### Incremental relations

Additional file 1: Tables S2 to S7 show the complete results of all assessments of incremental relations. Personality dysfunction was incrementally linked to drive for thinness (β = 0.25, *p* < 0.001, Δ*R*^2^ = 0.03), bulimia (β = 0.28, *p* < 0.001, Δ*R*^2^ = 0.03), body dissatisfaction (β = 0.30, *p* < 0.001, Δ*R*^2^ = 0.04), and binge eating (β = 0.35, *p* < 0.001, Δ*R*^2^ = 0.05), but not orthorexia (β = 0.07, Δ*R*^2^ = , *p* = 0.151, Δ*R*^2^ = 0.00) beyond the five ICD-11 personality trait domains.

Table 3 displays incremental relations of the ICD-11 personality trait domains with eating pathology beyond personality dysfunction. Negative affectivity statistically predicted all domains of eating pathology incrementally (β = 0.17 to 0.24, Δ*R*^2^ = 0.01 to 0.03, all *p*s < 0.001), except body dissatisfaction (β = 0.07, Δ*R*^2^ = 0.00, *p* = 0.111), beyond personality dysfunction. Detachment was negatively related to drive for thinness (β = −0.11, Δ*R*^2^ = 0.02, *p* = 0.001), bulimia (β = −0.11, Δ*R*^2^ = 0.01, *p* < 001.), and binge eating (β = −0.14, Δ*R*^2^ = 0.02, *p* < 0.001), after controlling for personality dysfunction. Dissociality did not display incremental relations with eating pathology beyond personality dysfunction (all *p*s ≥ 0.058). Disinhibition was positively related to bulimia (β = 0.11, Δ*R*^2^ = 0.02, *p* < 0.001) and binge eating (β = 0.11, Δ*R*^2^ = 0.01, *p* < 0.001), and negatively related to orthorexia (β = −0.15, Δ*R*^2^ = 0.02, *p* < 0.001), after controlling for personality dysfunction. Finally, anankastia was positively related to orthorexia beyond personality dysfunction (β = 0.18, Δ*R*^2^ = 0.03, *p* < 0.001).

**Table 3:** Incremental relations of the ICD-11 personality trait domains with eating pathology beyond personality dysfunction

ICD-11 personality trait domains	Drive for thinness		Bulimia		Body dissatisfaction		Orthorexia		Binge eating	
	β	Δ*R*^2^	β	Δ*R*^2^	β	Δ*R*^2^	β	Δ*R*^2^	β	Δ*R*^2^
Negative affectivity	0.24*	0.03*	0.19*	0.02*	0.07	0.00	0.21*	0.02*	0.17*	0.01*
Detachment	−0.11*	0.02*	−0.11*	0.01*	−0.03	0.00	−0.01	0.00	−0.14*	0.02*
Dissociality	0.00	0.00	0.04	0.01	−0.02	0.00	0.07	0.00	0.04	0.00
Disinhibition	−0.03	0.00	0.10*	0.01*	0.02	0.00	−0.15*	0.02*	0.11*	0.01*
Anankastia	0.06	0.01	−0.03	0.01	0.00	0.00	0.18*	0.03*	−0.05	0.00

β = standardized regression coefficient. Δ*R*^2^ = improvements in *R*^2^ compared to a model with only personality dysfunction as predictor. Additional file 1: Tables S2 to S7 display the complete results for all models. *p < 0.005

### Interactive relations

**Fig. 1:**
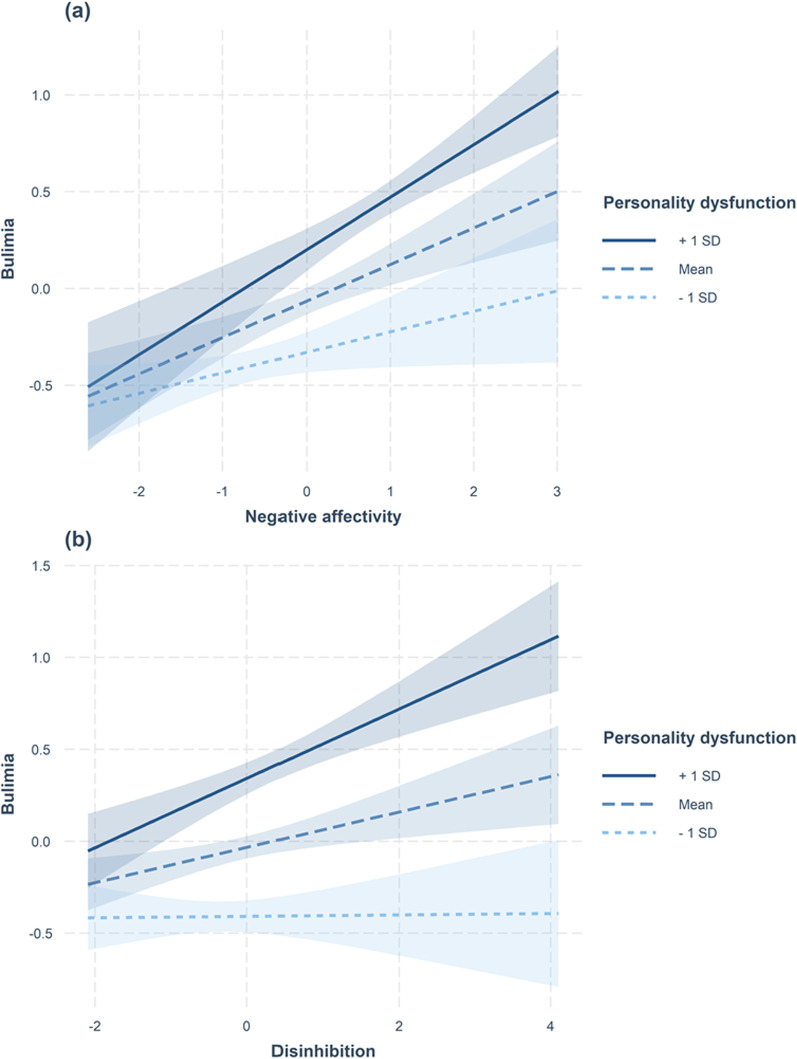
Relations of bulimia with **a** negative affectivity and **b** disinhibition for persons with high (+ 1 standard deviation), mean, or low (-1 standard deviation) personality dysfunction. *SD* = standard deviation. The shaded areas represent 95% confidence intervals

Additional file 1: Tables S2 to S7 also show the complete results for all tests of interactive relations. The interaction of personality dysfunction with negative affectivity (β = 0.08, Δ*R*^2^ = 0.01, *p* = 0.002) and disinhibition (β = 0.09, Δ*R*^2^ = 0.01, *p* < 0.001) predicted bulimia statistically significantly. As shown in Fig. 1, the associations of negative affectivity and disinhibition were more pronounced in persons with higher personality dysfunction than in persons with lower personality dysfunction. None of the other interaction terms reached statistical significance (all *p*s ≥ 0.086).

## Discussion

This study provided the first insights into relations between the ICD-11 personality disorder model and eating pathology. All eating pathology domains were robustly bivariately related to personality dysfunction. Also incrementally, personality dysfunction was remarkably strongly associated with all eating pathology domains, except for orthorexia. Hence, reduced intra- and interpersonal functioning may be central to eating pathology beyond trait-specific (e.g., anankastic) tendencies.

Regarding implications for psychotherapeutic treatment, this study’s results should be interpreted with great caution. All identified associations were cross-sectional and require replication in longitudinal designs. If these replications were successful, this might provide some preliminary cues that interventions targeting personality dysfunction (see [14, 62]) could constitute a promising approach for addressing eating pathology. Regarding theory building, contemporary models of eating pathology often encompass specific personality traits (e.g., perfectionism [29]). Following further replication of our findings, these models could be extended by incorporating *trait-general* personality dysfunction. A shared element of personality dysfunction contributes to the high comorbidity among personality disorders (see [15]). Thus, the consequences of experiencing persistent intra- and interpersonal difficulties might also explain the comorbidities of eating disorders with various *different* personality disorders (see [5]).

Negative affectivity was related to all eating pathology domains. This finding dovetails with previous notions of neuroticism as a transdiagnostic risk factor for eating pathology [2]. Regarding differential relations with eating pathology domains, negative affectivity was most weakly related to orthorexia, mirroring previous work on orthorexia and the DSM-5 AMPD [37]. Additionally, the incremental relations with drive for thinness, bulimia, orthorexia, and binge eating show that negative affectivity and eating pathology share unique manifestations beyond personality dysfunction.

Detachment showed only small and mostly not statistically-significant relations with eating pathology (β = 0.07 to 0.13). After controlling for personality dysfunction, a surprising pattern emerged: Detachment was related to lower drive for thinness, bulimia, and binge eating. The idea that, after partialling its problematic aspects, interpersonal and emotional distance (i.e., higher ICD-11 detachment) has adaptive components linked to lower eating pathology is difficult to reconcile with the current scientific literature. As detachment showed some small positive bivariate associations with eating pathology domains, its unique negative associations may also be a statistical artifact caused by the overlap of maladaptive aspects of detachment and personality dysfunction. Thus, this finding should be interpreted cautiously and must be replicated using different instrumentation and samples (e.g., clinical).

Dissociality was bivariately related to all and incrementally related to none of the eating pathology domains. Thus, the dissociality-eating pathology associations may be driven by the personality dysfunction component inherent to all maladaptive personality traits (see [19]). This finding is in line with previous research indicating that differences in emotional stability underlie differences in agreeableness between persons with and without eating disorders [63]. Together with this prior work, the current study highlights the importance of differentiating personality domain-specific and domain-general relations with eating pathology.

Disinhibition was, bivariately and incrementally, most strongly positively related to bulimia and binge eating. Thus, disinhibition appears to be uniquely linked to eating pathology characterized by a perceived loss of control. Whereas the link between binge eating and impulsivity is well-established (e.g., [33]), findings on associations between bulimia nervosa and impulsivity have been mixed (for a meta-analysis, see [64]). Further research is needed to establish whether studies using validated self-report instruments aligned with the ICD-11 personality disorder model and dimensional assessments of bulimia symptoms produce more consistent results than previous categorical approaches.

Anankastia was positively associated with eating pathology characterized by restricted eating (quantitatively as part of a drive for thinness or qualitatively as a part of orthorexic fixation on health-conscious eating). Additionally, anankastia was incrementally linked to orthorexia beyond personality dysfunction. This pattern of results dovetails with previously identified relations of obsessive–compulsive traits, such as perfectionism, with anorexia [65] and orthorexia [35]. In previous studies, perfectionism has also been linked to body dissatisfaction [34] and binge eating [66]. As these associations were not observed for the anankastia scale, this study strengthens the argument that ICD-11 anankastia may not capture all maladaptive elements of perfectionism [67].

The tests of interactive effects of personality trait domains and personality dysfunction on eating pathology showed that the personality trait domain-eating pathology association did not depend on the severity of personality dysfunction. Two statistically significant interactions provided some preliminary evidence that the relations of negative affectivity and disinhibition with bulimia are more pronounced in persons with high personality dysfunction. Yet, this pattern was not observed for all other personality—and eating pathology domains (i.e., 92% of the investigated interactions). Thus, this study showed that an initial screening of personality dysfunction (see [9]) is not required to identify persons in which the ICD-11 personality trait domains are related to eating pathology.

### Limitations and future research

This study has several limitations. First, it requires replication using different samples. For instance, the associations may differ in populations with more pronounced personality or eating disturbances (i.e., clinical samples). For example, the EDI-2 bulimia subscale may assess uncontrolled overeating rather than clinical symptoms of bulimia nervosa in general population samples. In our study, we did not assess whether participants were diagnosed with an eating disorder. However, the observed eating pathology measures’ mean scores were higher than previously reported scores in healthy controls (e.g., [51]). This indicates that persons with eating disturbances might be represented overproportionally in our study (e.g., due to an increased interest of affected persons in eating pathology studies).

Second, it is unclear to what extent shared method bias contributed to our findings. Hence, future research using clinician reports of personality and eating pathology is needed. Third, we did not assess the borderline pattern qualifier that has been added belatedly to the ICD-11 personality disorder model following controversial discussion [11]. Borderline personality disorder is frequently associated with eating pathology (e.g., [2]). Hence, future work is needed to test bivariate, incremental, and interactive effects of this additional ICD-11 personality disorder component with disturbed eating. Fourth, our design was cross-sectional. Thus, future longitudinal research is needed to clarify causal links of the ICD-11 personality disorder model components with eating pathology and the model’s utility for informing the treatment of persons with eating pathology. Additionally, further work is needed to clarify the unique utility of personality dysfunction and maladaptive traits for different contexts and outcomes beyond eating pathology (see [16, 39]).

## Conclusions

This study showed that the components of the ICD-11 personality disorder are meaningfully and differentially associated with different eating pathology domains on the bivariate level. Additionally, personality dysfunction and most ICD-11 personality trait domains were incrementally and independently linked to eating pathology. Taken together, these findings indicate that the ICD-11 personality trait domains and personality dysfunction are complementary—and not redundantly—associated with eating pathology. Thus, this study provides preliminary arguments that both components of the ICD-11 personality disorder model should routinely be assessed.

## Supplementary Information

**Additional file 1.** Supplementary statistical information.

## Data Availability

The data and R scripts are available via the Open Science Framework (OSF): https://osf.io/ybwu9/?view_only=81f7f8794bbf438790c5e58ab829794b.
