# Supplementary material for: An exploratory study of associations between the ICD-11 personality disorder model and eating pathology
Source: J Eat Disord. 2022 Aug 31;10:130. doi: 10.1186/s40337-022-00658-y (PMC9429753; doi:10.1186/s40337-022-00658-y)
Supplement: Supplementary file 1 — Additional file 1. Supplementary statistical information. [file 40337_2022_658_MOESM1_ESM.pdf]

Supplementary Information

An Exploratory Study of Associations Between the ICD-11 Personality Disorder Model and Eating Pathology

Table S1

Bivariate Correlations, Means (M), Standard Deviations (SD), and Internal Consistencies for all Variables

Variable	1	2	3	4	5	6	7	8	9	10	11
1. Personality dysfunction	-										
2. Negative affectivity	.71*	-									
3. Detachment	.44*	.35*	-								
4. Dissociality	.34*	.20*	.19*	-							
5. Disinhibition	.33*	.22*	.15*	.39*	-						
6. Anankastia	.20*	.40*	.30*	-.05	-.46*	-					
7. Drive for thinness	.37*	.38*	.07	.12*	.10*	.13*	-				
8. Bulimia	.42*	.39*	.10*	.18*	.24*	.05	.65*	-			
9. Body dissatisfaction	.33*	.27*	.13*	.09*	.13*	.07	.71*	.49*	-		
10. Orthorexia	.19*	.24*	.08	.13*	-.07	.21*	.53*	.36*	.20*	-	
11. Binge eating	.46*	.41*	.09	.19*	.25*	.04	.72*	.82*	.62*	.32*	-
M	24.24	34.44	26.56	24.21	25.76	39.11	21.19	13.83	32.51	18.13	27.85
SD	6.31	8.17	8.00	6.35	6.58	6.66	8.92	6.19	12.04	5.50	8.93
Cronbach's α	.85	.85	.85	.78	.78	.79	.91	.88	.93	.91	.84

Note. $^*p < .005$.

Table S2

Results From Five Hierarchical Regressions With the ICD-11 Personality Trait Domains and Personality Dysfunction as Predictors of Eating Pathology

	Drive for thinness			Bulimia			Body dissatisfaction			Orthorexia			Binge eating		
Variables	β	R^2	ΔR^2	β	R^2	ΔR^2	β	R^2	ΔR^2	β	R^2	ΔR^2	β	R^2	ΔR^2
Step 1		.15			.19			.08			.10			.20	
Negative affectivity	.39*			.37*			.26*			.23*			.41*		
Detachment	-.08			-06			.04			-.03			-.07		
Dissociality	.06			.06			.02			.14*			.07		
Disinhibition	.00			.13*			.04			-.14*			.12*		
Anankastia	.00			-.02			-.03			.07			-.04		
Step 2		.18	.03*		.22	.03*		.12	.04*		.10	.00		.25	.05*
Negative affectivity	.24*			.20*			.07			.18*			.19*		
Detachment	-.13*			-.12*			-.03			-.04			-.14*		
Dissociality	.02			.02			-.03			.13*			.02		
Disinhibition	-.01			.11			.02			-.15*			.10		
Anankastia	.02			.00			-.01			.07			-.02		
Personality dysfunction	.25*			.28*			.30*			.07			.35*		

Note. β = standardized regression coefficient. *p < .005.

Table S3

Results From Five Hierarchical Regressions With Negative Affectivity and Personality Dysfunction as Predictors of Eating Pathology

	Drive for thinness			Bulimia			Body dissatisfaction			Orthorexia			Binge eating		
Variables	β	R^2	ΔR^2	β	R^2	ΔR^2	β	R^2	ΔR^2	β	R^2	ΔR^2	β	R^2	ΔR^2
Step 1		.13			.17			.11			.04			.21	
Personality dysfunction	.37*			.41*			.34*			.20*			.46*		
Step 2		.16	.03*		.19	.02*		.11	.00		.06	.02*		.22	.01*
Personality dysfunction	.20*			.27*			.29*			.04			.34*		
Negative affectivity	.24*			.19*	.		.07			.21*			.17*		
Step 3		.16	.00		.20	.01*		.11	.00		.06	.00		.23	.00
Personality dysfunction	.20*			.27*			.29*			.05			.33*		
Negative affectivity	.24*			.19*			.07			.22*			.17*		
Personality dysfunction* negative affectivity	.01			.08*			.02			-.03			.05		

Note. β = standardized regression coefficient. $^*p < .005$.

Table S4

Results From Five Hierarchical Regressions With Detachment and Personality Dysfunction as Predictors of Eating Pathology

	Drive for thinness			Bulimia			Body dissatisfaction			Orthorexia			Binge eating		
Variables	β	R^2	ΔR^2	β	R^2	ΔR^2	β	R^2	ΔR^2	β	R^2	ΔR^2	β	R^2	ΔR^2
Step 1		.13			.17			.11			.04			.21	
Personality dysfunction	.37*			.41*			.34*			.20*			.46*		
Step 2		.15	.02*		.18	.01*		.11	.00		.04	.00		.23	.02*
Personality dysfunction	.41*			.46*			.35*			.20*			.52*		
Detachment	-.11*			-.11*			-.03			-.01			-.14*		
Step 3		.15	.00		.18	.00		.11	.00		.04	.00		.23	.00
Personality dysfunction	.42*			.46*			.35*			.20*			.52*		
Detachment	-.11*			-.11*			-.03			-.01			-.13*		
Personality dysfunction* detachment	-.01			.01			.01			.00			-.02		

Note. β = standardized regression coefficient. *$p < .005$.

Table S5

Results From Five Hierarchical Regressions With Dissociality and Personality Dysfunction as Predictors of Eating Pathology

	Drive for thinness			Bulimia			Body dissatisfaction			Orthorexia			Binge eating		
Variables	β	R^2	ΔR^2	β	R^2	ΔR^2	β	R^2	ΔR^2	β	R^2	ΔR^2	β	R^2	ΔR^2
Step 1		.13			.17			.11			.04			.21	
Personality dysfunction	.37*			.41*			.34*			.20*			.46*		
Step 2		.13	.00		.18	.01		.11	.00		.04	.00		.21	.00
Personality dysfunction	.37*			.40*			.34*			.17*			.45*		
Dissociality	.00			.04			-.02			.07			.04		
Step 3		.13	.00		18	.01		.11	.00		.04	.00		.21	.00
Personality dysfunction	.37*			.40*			.34*			.17*			.45*		
Dissociality	-.01			.04			-.02			.06			.04		
Personality dysfunction* dissociality	.01			.00			.00			.04			-.03		

Note. β = standardized regression coefficient. $^*p < .005$.

Table S6

Results From Five Hierarchical Regressions With Disinhibition and Personality Dysfunction as Predictors of Eating Pathology

	Drive for thinness			Bulimia			Body dissatisfaction			Orthorexia			Binge eating		
Variables	β	R^2	ΔR^2	β	R^2	ΔR^2	β	R^2	ΔR^2	β	R^2	ΔR^2	β	R^2	ΔR^2
Step 1		.13			.17			.11			.04			.21	
Personality dysfunction	.37*			.41*			.34*			.20*			.46*		
Step 2		.14	.01		.19	.02*		.11	.00		.06	.02*		.22	.01*
Personality dysfunction	.38*			.37*			.33*			.25*			.42*		
Disinhibition	-.02			.11*			.02			-.15*			.11*		
Step 3		.14	.00		.20	.01*		.11	.00		.06	.00		.22	.00
Personality dysfunction	.38*			.38*			.33*			.24*			.42*		
Disinhibition	-.03			.10*			.01			-.14*			.11*		
Personality dysfunction* disinhibition	.01			.09			.03			-.04			.03		

Note. β = standardized regression coefficient. *$p < .005$.

Table S7

Results From Five Hierarchical Regressions With Anankastia and Personality Dysfunction as Predictors of Eating Pathology

	Drive for thinness			Bulimia			Body dissatisfaction			Orthorexia			Binge eating		
Variables	β	R^2	ΔR^2	β	R^2	ΔR^2	β	R^2	ΔR^2	β	R^2	ΔR^2	β	R^2	ΔR^2
Step 1		.13			.17			.11			.04			.21	
Personality dysfunction	.37*			.41*			.34*			.20*			.46*		
Step 2		.14	.01		.18	.01		.11	.00		.07	.03*		.21	.00
Personality dysfunction	.36*			.42*			.34*			.16*			.47*		
Anankastia	.06			-.03			.00			.18*			-.05		
Step 3		.14	.00		.18	.00		.11	.00		.07	.00		.21	.00
Personality dysfunction	.35*			.42*			.34*			.16*			.47*		
Anankastia	.06			-.03			.00			.18*			-.05		
Personality dysfunction* anankastia	.01			.01			.00			.00			.01		

Note. β = standardized regression coefficient. $^*p < .005$.
